# Fast-killing parasites can be favoured in spatially structured populations

**DOI:** 10.1098/rstb.2016.0096

**Published:** 2017-03-13

**Authors:** Helen C. Leggett, Geoff Wild, Stuart A. West, Angus Buckling

**Affiliations:** 1 Department of Genetics, University of Cambridge, Cambridge CB2 3EH, UK; 2 Biosciences, University of Exeter, Cornwall Campus, Penryn TR10 9FE, UK; 3 Department of Applied Mathematics, University of Western Ontario, London, Ontario, Canada N6A 5B7; 4 Department of Zoology, University of Oxford, Oxford OX1 3PS, UK

**Keywords:** transmission, parasites, growth, virulence, population structure

## Abstract

It is becoming increasingly clear that the evolution of infectious disease is influenced by host population structure. Theory predicts that parasites should be more ‘prudent’—less transmissible—in spatially structured host populations. However, here we (i) highlight how low transmission, the phenotype being selected for in this in context, may also be achieved by rapacious host exploitation, if fast host exploitation confers a local, within-host competitive advantage and (ii) test this novel concept in a bacteria–virus system. We found that limited host availability and, to a lesser extent, low relatedness favour faster-killing parasites with reduced transmission. By contrast, high host availability and high relatedness favour slower-killing, more transmissible parasites. Our results suggest high, rather than low, virulence may be selected in spatially structured host–parasite communities where local competition and hence selection for a within-host fitness advantage is high.

This article is part of the themed issue ‘Opening the black box: re-examining the ecology and evolution of parasite transmission’.

## 1. Introduction

Owing to the importance of infectious diseases to human health, agriculture and wildlife populations [1–7], it is critical that we better understand how ecology shapes the evolution of life histories of infectious organisms. In particular, host spatial population structure is ubiquitous in natural and managed populations. A growing body of theoretical models, observational studies and experiments suggests that host population structure will select for ‘prudent’ parasites with lower rates of parasite transmission, because this strategy leads to more-efficient exploitation of the susceptible local host population and higher parasite densities. A reason for evolution towards lower transmission rate in structured populations is due to local extinctions of infected hosts: a higher transmission rate is selected until a critical point is reached beyond which any further increase in transmission will cause the local cluster of hosts to be wiped out very rapidly [8]. In addition to the direct fitness benefit of low transmission in structured host populations, there is also an indirect fitness benefit: host population structure increases parasite relatedness [9,10], hence low transmission reduces competition between kin [9,11–19].

The above models [9,11–15], and many others [20–25], assume that transmission is a positive function of within-host growth rate, which also affects pathogen virulence (reductions in host fitness caused by the parasite). Natural selection will favour an optimal level of virulence that tends to maximize parasite transmission rate and minimize infected-host death rate because death of the host ceases transmission [9,21,26]. Low transmission can therefore result from both high and low within-host growth rate and hence virulence. However, while mild parasites do not regulate the host population, high virulence may have negative consequences on host availability if infected hosts die and/or recovered hosts cannot reproduce; hence models typically predict that population structure will select for low transmission and low virulence [8].

Many parasites, however, must kill their hosts to achieve transmission, and in these contexts selection by definition favours maximal virulence. These obligately killing parasites are found among many groups of organisms, including bacteriophages, nuclear polyhedroviruses, bacteria, nematodes, fungi and microsporidia [27]. They grow inside their host and convert host biomass into parasite transmission stages that are released into the environment when the host ruptures [28,29]. However, obligate killing parasites face an analogous virulence-transmission trade-off to parasites that need to transmit from live hosts, in terms of the rate they kill their hosts. Specifically, some intermediate rate of kill will maximize transmission; too short, and there will be fewer propagules developed inside the host before host death, and too long inevitably reduces the rate of transmission [27]. Crucially, in the case of obligate killing parasites, infected hosts are unlikely to have opportunities to reproduce before they are killed, hence in principle, selection for low transmission could be achieved by fast and slow killing.

The optimal levels of parasite virulence and time to kill will be affected by a range of ecological factors, including extrinsic host mortality, host immune status and competition with other parasite genotypes [27,30,31]. This latter selection pressure is likely to be particularly relevant in structured populations, because limited host availability may influence the likelihood of coinfection of competing parasite genotypes. Assuming competition between coinfecting parasites is mediated purely by resource competition (as opposed to, e.g. interference competition or intraspecific exploitation of public goods [32–34]), then coinfection tends to favour higher growth rates (and hence higher virulence or faster time to kill) to maximize within-host competitive ability [18,20,35,36]. If fast and slow killing have the same consequences for disease transmission, the added advantage of increased within-host competitive ability may, in some contexts, favour faster time to kill in structured host populations.

Here, we investigate if (i) faster killing can be favoured over slow killing under conditions where there is limited local host availability, and (ii) whether selection for time to kill is altered by within-host competition between parasite genotypes, using an obligately killing virus and its bacterial host. We selected on standing variation in time to kill from viruses evolved in a previous study, in which we showed that faster-killing viruses are more competitive within hosts but have lower transmission [36]. To allow us to independently manipulate local host availability and relatedness, both of which will be affected by host spatial structure, we carried out selection in experimental metapopulations, where hosts were limited or not and single or multiple viral clones were inoculated into each subpopulation (high and low relatedness).

## 2. Material and methods

### (a) Experimental viral strains

We previously evolved 12 populations of an obligately killing virus (bacteriophage ϕ2) on its host bacterium *Pseudomonas fluorescens* for 300 generations, under conditions of low or variable multiplicity of infection (MOI), six lines evolved under each treatment. Virus evolved under variable MOI killed host cells faster in multiple infections, at the cost of reduced transmission to new hosts. Virus evolved under low MOI had a slower time to kill in multiple infections, resulting in lower within-host competitiveness but increased transmission [36]. We isolated a single clone from each experimental population to be used in subsequent experiments.

### (b) Selection experiment

We evolved 24 metapopulations of ϕ2 virus on *P. fluorescens* for three transfers, approximately 20 phage generations. This short time scale was sufficient for the experiment as we were selecting on large amounts of standing variation from our previously evolved viral populations, rather than relying on de novo mutation. Each metapopulation consisted of 12 subpopulations (wells in microtitre plates) containing 200 µl Kings Media B (KB) and 10^2^ exponentially growing ancestral bacteria. It was necessary to establish high MOI in all treatments because our experimental viruses only kill host cells rapidly during multiple infections [36]. Thus, we inoculated each patch with 10^8^ viruses. We previously demonstrated that coinfections readily occur in this system under these conditions by using marked phages [36,37].

**Figure 1.**
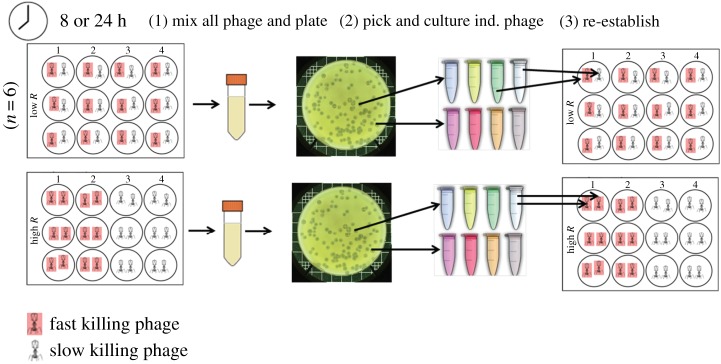
Experimental set-up. We manipulated viral relatedness and host availability in a fully factorial design. We started the experiment with diverse metapopulations of fast- and slow-killing phage. Half the metapopulations were relatively high relatedness treatments—subpopulations (wells in microtitre plates) founded with single phage clones, while half were relatively low relatedness—founded with two clones. We manipulated host availability by varying the amount of time host-virus populations were cultured: 24 versus 8 h; hosts were a more or less limited resource, meaning that parasite growth was relatively constrained and unconstrained, respectively. (1) At each transfer, we mixed together all subpopulations within a metapopulation and (2) randomly selected virus clones from the plated mixture to (3) re-establish new subpopulations: the output of a subpopulation was consequently dependent on its productivity (global competition).

Each metapopulation was established with 12 virus subpopulations that varied in their time to kill (described above, fast: 28.69 ± 0.61 min, or slow: 36.54 ± 0.32 min). Six replicate metapopulations were founded with single (and unique) viral clones in each subpopulation (high relatedness (genetically identical) treatments), while six metapopulations had 12 individual subpopulations founded with one fast-killing clone and one slow-killing clone, with each of the 12 viral clones used twice per replicate metapopulation (low relatedness (genetically non-identical) treatments). To manipulate host availability, half of the metapopulations were transferred every 8 h and the other half of the metapopulations were transferred every 24 h. In both cases, hosts were a limiting resource, given the high MOI, but lower growth rates over 24 h than 8 h during preliminary work suggests that the former results in greater host limitation. The experimental design is shown in figure 1.

In each transfer, we combined all the subpopulations within a metapopulation to simulate global competition within spatially structured populations, by thoroughly mixing the entire metapopulation in a 10 ml falcon tube, treating with 10% chloroform and centrifuging at 14 000 r.p.m. for 2 min to isolate the phage, then isolated individual phage clones by plating dilutions of phage populations onto KB agar plates with a semisolid overlay *P. fluorescens* lawn. We then picked individual clones (phage plaques) with sterile pipette tips, amplified them overnight in liquid KB media plus 10^6^ ancestral *P. fluorescens* so they reached the same densities, then redistributed 10^8^ phage into a fresh set of wells containing 10^2^ bacteria to reestablish the starting treatment conditions. There were four treatments in total: (i) low relatedness and high host availability, (ii) low relatedness and low host availability, (iii) high relatedness and high host availability and (iv) high relatedness and low host availability (figure 1). At the end of the selection, we assessed the mean lysis time (*t*_50_) of each of the populations of virus from each of the four treatments.

### (c) Measuring time to kill

We measured the population-level time taken for viruses to lyse bacteria cells in each of our 24 metapopulations in a ‘one-step growth experiment’ [38]. We added 10^8^ phage to 10^2^ exponentially growing bacteria in 20 µl KB media and measured phage density by plating onto bacterial lawns at time zero and then at 5-min intervals from 25 min (we never observed increases in phage density prior to 30 min in our previous studies) [36]. Our specific measure of lysis time was *t*_50_, the number of minutes taken to reach 50% of maximal phage density during a single synchronized growth cycle.

### (d) Statistical analysis

We analysed variation in phage growth as a factorial generalized linear model (GLM) with explanatory variable ‘relatedness’ (high or low) and ‘time’ (8 or 24) and their interaction. We analysed variation in phage population time to kill (*t*_50_) as a factorial GLM with explanatory variables ‘relatedness’ (high or low) and ‘host availability’ (unlimited or limited) and their interaction. We conducted pairwise comparisons of *t*_50_ with Tukey's pairwise comparison tests using function ‘glht’ in package ‘multcomp’ [39] in R. We carried out all analyses and drew all figures using R v. 2.15.3.

## 3. Results and discussion

**Figure 2.**
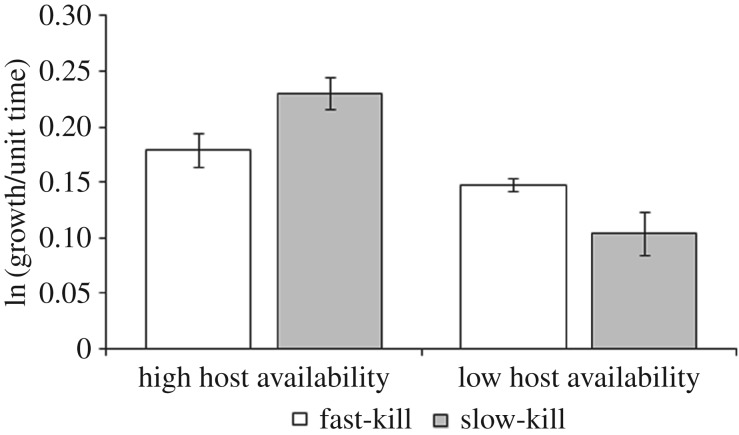
Mean population growth (multiplication rate, logged) per unit time (hour), of fast- and slow-killing virus strains. Over 8 h, host availability was less limited, resulting in higher relative population growth rate of the higher transmitting and less virulent (slow-killing) populations, whereas hosts were more limiting over 24 h, resulting in higher population growth of the lower transmitting and virulent (fast-killing) populations. Data are means (*n* = 6) ± s.e.m.

We started the experiment with viruses we had previously evolved to vary in their time to kill. Faster-killing viruses have greater within-host competitive ability, but lower between-host transmission [36]. We first wanted to determine whether the lower transmission of the faster-killing viruses resulted in a higher net growth rate than that of the more transmissible, slower-killing virus when there is limited host availability, and vice versa when hosts are abundant. To do this, we assayed the relative growth rate of populations of the experimental viruses over 8 and 24 h when host availability is relatively unlimited and limited, respectively. As predicted, (i) at 8 h when parasite growth is relatively unconstrained by host availability, the slower-killing viruses had a higher growth rate (figure 2) and (ii) at 24 h when parasite growth is constrained by limited host availability, the faster-killing populations had a higher growth rate (figure 2, significant effect of time × strain interaction *F*_1,20_ = 7.84, *p* = 0.012).

**Figure 3.**
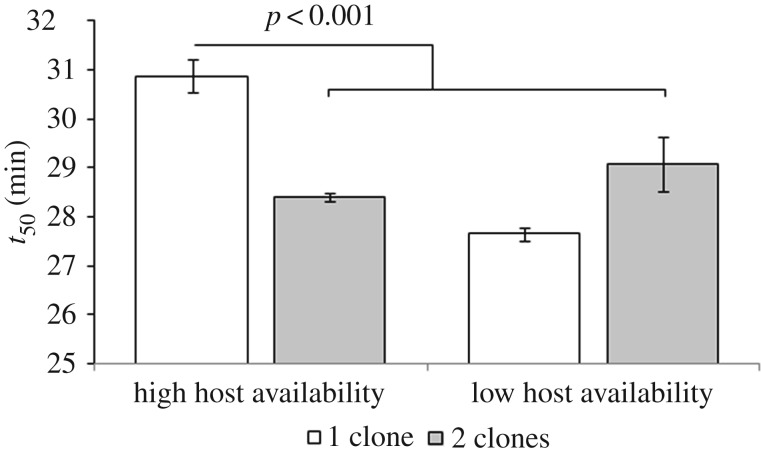
Time to kill (*t*_50_) of evolved virus populations. Viruses were evolved in subpopulations where relatedness was either relatively high (1 clone) or low (2 clones), and where their growth was relatively unconstrained or constrained by high or low host availability. Data are means (*n* = 6) ± s.e.m.

We next wanted to determine how competition between genotypes and host availability might interact to determine the relative success of faster-killing versus slower-killing viruses, given that both relatedness and host availability might be affected by spatial structure. To allow us to independently manipulate local host availability and competition between genotypes, we carried out selection in experimental metapopulations, where hosts were more or less limited and single or two viral clones (one fast and slow killing) were inoculated into each patch (relatedness is relatively high or low; *r* = 1 or *r* < 1). Note that given the high ratio of viruses to hosts, high and low relatedness refers to both within-subpopulation and within-host scales. We found an interaction between host availability and relatedness, such that when host availability was limited, fast time to kill (approx. 28 min) is favoured regardless of relatedness; whereas when host availability was unlimited and relatedness was high, time to kill was significantly slower (approx. 31 min; figure 3, significant relatedness × host availability: *F*_1,14_ = 37.82, *p* < 0.001, Tukey's pairwise comparisons, *p* < 0.001).

Consistent with previous correlative and experimental studies [16–19], our experiment shows that limited host availability in spatially structured environments selects for prudent, slow-transmitting parasites. Most theory and data suggest that this also results in low virulence/slow time to kill for obligate killers; but here we show low transmission resulting from fast-killing viruses. It is important to emphasize that our experimental design (specifically, selection on standing variation) has constrained the outcome to lower transmission being achieved by faster time to kill. Indeed, previous experimental work demonstrating that parasites can evolve to have lower transmission when infection occurs locally [16] reported a correlated reduction in competitive ability, which is consistent with a slower rather than faster time to kill. The other experimental study reporting a similar finding only measured infectivity and not correlated life-history traits [17]. However, our results suggest that selection for low transmission may favour faster killing as opposed to slow, because fast or slow killing can achieve the same optimal level of transmission. Analogous arguments can be applied to the evolution of high and low virulence strategies if the strategies have equivalent impacts on parasite transmission and host population growth rate, which may be the case if infected hosts are unable to reproduce following infection and recovery.

In addition to the direct fitness benefit of low transmission being a more-efficient host exploitation strategy in viscous populations, there is an indirect fitness benefit that low transmission reduces competition between kin [15]. We show both processes can have independent and important effects, although host availability appears to be more significant than relatedness in this case. As within our previous study [36], we found that faster time to kill was favoured in mixed versus single clone (low versus high relatedness) infections where parasite growth is less constrained. However, when growth is more constrained by host availability, relatedness had no effect: fast killing was always selected. These results highlight how consequences of population structure, in this case local host availability, can interact in their effect on parasite life-history evolution. In summary, our key result is that fast-killing parasites can be selected in spatially structured populations because, like slow-killing parasites, they are less transmissible and hence use their hosts ‘prudently’.

## Supplementary Material

Raw data

## Data accessibility

The datasets supporting this article have been uploaded as part of the electronic supplementary material.

## References

[1] Keeling MJ et al. 2001 Dynamics of the 2001 UK foot and mouth epidemic: stochastic dispersal in a heterogeneous landscape. Science 294, 813–817. (10.1126/science.1065973) 11679661

[2] Boots M, Best A, Miller MR, White A 2009 The role of ecological feedbacks in the evolution of host defence: what does theory tell us? Phil. Trans. R. Soc. B 364, 27–33. (10.1098/rstb.2008.0160) 18930880 PMC2666696

[3] Lloyd-Smith JO, Schreiber SJ, Kopp PE, Getz WM 2005 Superspreading and the effect of individual variation on disease emergence. Nature 438, 355–359. (10.1038/nature04153) 16292310 PMC7094981

[4] Hudson PJ, Dobson AP, Newborn D 1998 Prevention of population cycles by parasite removal. Science 282, 2256–2258. (10.1126/science.282.5397.2256) 9856948

[5] Tompkins DM, White AR, Boots M 2003 Ecological replacement of native red squirrels by invasive greys driven by disease. Ecol. Lett. 6, 189–196. (10.1046/j.1461-0248.2003.00417.x)

[6] Haydon DT et al. 2006 Low coverage vaccination strategies for the conservation of endangered species. Nature 443, 692–695. (10.1038/nature05177) 17036003

[7] Lafferty KD et al. 2008 Parasites in food webs: the ultimate missing links. Ecol. Lett. 11, 533–546. (10.1111/j.1461-0248.2008.01174.x) 18462196 PMC2408649

[8] Lion S, Boots M 2010 Are parasites ‘prudent’ in space? Ecol. Lett. 13, 1245–1255. (10.1111/j.1461-0248.2010.01516.x) 20727004 PMC3070161

[9] Frank SA 1996 Models of parasite virulence. Q. Rev. Biol. 71, 37–78. (10.1086/419267) 8919665

[10] Queller DC 1994 Genetic relatedness in viscous populations. Evol. Ecol. 8, 70–73. (10.1007/BF01237667)

[11] Rand DA, Keeling M, Wilson HB 1995 Invasion, stability and evolution to criticality in spatially extended, artificial host-pathogen ecologies. Proc. R. Soc. Lond. B 259, 55–63. (10.1098/rspb.1995.0009)

[12] Boots M, Sasaki A 1999 Small worlds’ and the evolution of virulence: infection occurs locally and at a distance. Proc. R. Soc. Lond. B 266, 1933–1938. (10.1098/rspb.1999.0869) PMC1690306 10584335

[13] Haraguchi Y, Sasaki A 2000 The evolution of parasite virulence and transmission rate in a spatially structured population. J. Theor. Biol. 203, 85–96. (10.1006/jtbi.1999.1065) 10704294

[14] Kamo M, Sasaki A, Boots M 2006 The role of trade-off shapes in the evolution of parasites in spatial host populations: an approximate analytical approach. J. Theor. Biol. 244, 588–596. (10.1016/j.jtbi.2006.08.013) 17055535

[15] Wild G, Gardner A, West SA 2009 Adaptation and the evolution of parasite virulence in a connected world. Nature 459, 983–986. (10.1038/nature08071) 19474791

[16] Kerr B, Neuhauser C, Bohannan BJM, Dean AM 2006 Local migration promotes competitive restraint in a host-pathogen 'tragedy of the commons’. Nature 442, 75–78. (10.1038/nature04864) 16823452

[17] Boots M, Mealor M 2007 Local interactions select for lower pathogen infectivity. Science 315, 1284–1286. (10.1126/science.1137126) 17332415

[18] Herre EA 1993 Population structure and the evolution of virulence in nematode parasites of fig wasps. Science 259, 1442–1445. (10.1126/science.259.5100.1442) 17801279

[19] Ewald PW 1994 Evolution of infectious diseases. Oxford, UK: Oxford University Press.

[20] Levin S, Pimentel D 1981 Selection of intermediate rates of increase in parasite-host systems. Am. Nat. 117, 308–315. (10.1086/283708)

[21] Anderson RM, May RM 1982 Coevolution of hosts and parasites. Parasitology 85, 411–426. (10.1017/S0031182000055360) 6755367

[22] Levin BR et al. 1982 Evolution of hosts and parasites. In Population biology of infectious disease (eds Anderson RM, May RM). Berlin, Germany: Springer.

[23] May RM, Anderson RM 1983 Epidemiology and genetics in the coevolution of parasites and hosts. Proc. R. Soc. Lond. B 219, 281–313. (10.1098/rspb.1983.0075) 6139816

[24] de Roode JC, Yates AJ, Altizer S 2008 Virulence-transmission trade-offs and population divergence in virulence in a naturally occurring butterfly parasite. Proc. Natl Acad. Sci. USA 105, 7489–7494. (10.1073/pnas.0710909105) 18492806 PMC2396697

[25] Read JM, Keeling MJ 2003 Disease evolution on net-works: the role of contact structure. Proc. R. Soc. Lond. B 270, 699–798. (10.1098/rspb.2002.2305) PMC1691304 12713743

[26] Bremermann HJ, Thieme HR 1989 A competitive exclusion principle for pathogen virulence. J. Math. Biol. 27, 179–190. (10.1007/BF00276102) 2723551

[27] Ebert D, Weisser WW 1997 Optimal killing for obligate killers: the evolution of life histories and virulence of semelparous parasites. Proc. R. Soc. Lond. B 264, 985–991. (10.1098/rspb.1997.0136) PMC1688549 9263465

[28] Anderson RM, May RM 1981 The population dynamics of microparasites and their invertebrate hosts. Phil. Trans. R. Soc. Lond. B 291, 451–524. (10.1098/rstb.1981.0005)

[29] Miller LK, Lingg AJ, Bulla LA 1983 Bacterial, viral and fungal insecticides. Science 219, 715–721. (10.1126/science.219.4585.715) 17814032

[30] Bonhoffer S, Nowak MA 1994 Intra-host and inter-host selection: viral evolution of immune function impairment. Proc. Natl Acad. Sci. USA 91, 8062–8066. (10.1073/pnas.91.17.8062) 8058757 PMC44545

[31] van Baalen M, Sabelis MW 1995 The dynamics of multiple infection and the evolution of virulence. Am. Nat. 1446, 881–970. (10.1086/285830)

[32] Brown SP, Hochberg ME, Grenfell BT 2002 Does multiple infection select for raised virulence? Trends Microbiol. 10, 401–405. (10.1016/S0966-842X(02)02413-7) 12217504

[33] West SA, Buckling A 2003 Cooperation, virulence and siderophore production in bacterial parasites. Proc. R. Soc. Lond. B 270, 37–44. (10.1098/rspb.2002.2209) PMC1691207 12590769

[34] Gardner A, West SA, Buckling A 2004 Bacteriocins, spite and virulence. Proc. R. Soc. Lond. B 271, 1529–1535. (10.1098/rspb.2004.2756) PMC1691756 15306326

[35] Nowak MA, May RM 1994 Superinfection and the evolution of parasite virulence. Proc. R. Soc. Lond. B 255, 81–89. (10.1098/rspb.1994.0012) 8153140

[36] Leggett HC, Benmayor R, Hodgson DJ, Buckling A 2013 Experimental evolution of adaptive phenotypic plasticity in a parasite. Curr. Biol. 23, 1–4. (10.1016/j.cub.2012.11.045) 23246405

[37] Hall AR, Scanlan PD, Leggett HC, Buckling A 2012 Multiplicity of infection does not accelerate infectivity evolution of viral parasites in laboratory microcosms. J. Evol. Biol. 25, 409–415. (10.1111/j.1420-9101.2011.02434.x) 22168551

[38] Ellis EL, Delbrück M 1939 The growth of bacteriophage. J. Gen. Physiol. 22, 365–384. (10.1085/jgp.22.3.365) 19873108 PMC2141994

[39] Hothorn T, Bretz F, Westfall P 2008 Simultaneous inference in general parametric models. Biom. J. 50, 346–363. (10.1002/bimj.200810425) 18481363

