# Supplementary material for: Fast-killing parasites can be favoured in spatially structured populations
Source: Philos Trans R Soc Lond B Biol Sci. 2017 Mar 13;372(1719):20160096. doi: 10.1098/rstb.2016.0096 (PMC5352822; doi:10.1098/rstb.2016.0096)
Supplement: Raw data [file rstb20160096supp1.pdf]

Supplementary material for [Leggett et al.], [2016], [Fast-killing parasites can be favoured in spatially structured populations], *Phil. Trans. R. Soc. B.* doi: 10.1098/rstb.[paper ID in form xxxx.xxxx e.g. 10.1098/rstb.2014.0049]

Raw data for Figure 2

Strains	replicate	time (h)	dilution	count/20ul	density/ml	growth (ln)
HH	A	0	100000	72	360000000	
HH	B	0	100000	82	410000000	
HH	C	0	100000	75	375000000	
HH	D	0	100000	63	315000000	
LL	A	0	100000	84	420000000	
LL	B	0	100000	68	340000000	
LL	C	0	100000	68	340000000	
LL	D	0	100000	84	420000000	
HH	A	8	10000000	2	1000000000	1.021651
HH	B	8	1000000	25	1250000000	1.114742
HH	C	8	1000000	36	1800000000	1.568616
HH	D	8	10000000	3	1500000000	1.560648
HH	E	8	1000000	36	1800000000	1.595645
HH	F	8	1000000	42	2100000000	1.749795
LL	A	8	1000000	38	1900000000	1.509354
LL	B	8	10000000	6	3000000000	2.177422
LL	C	8	10000000	4	2000000000	1.771957
LL	D	8	1000000	45	2250000000	1.678431
LL	E	8	10000000	7	3500000000	2.220347
LL	F	8	1000000	41	2050000000	1.685424
HH	A	24	10000000	14	7000000000	3.506558
HH	B	24	10000000	44	22000000000	2.995732
HH	C	24	10000000	10	5000000000	3.912023
HH	D	24	10000000	22	11000000000	3.575551
HH	E	24	10000000	4	2000000000	3.935013
HH	F	24	1000000	62	3100000000	3.352407
LL	A	24	100000	94	470000000	1.324419
LL	B	24	100000	162	810000000	0.993252
LL	C	24	1000000	127	6350000000	3.745575
LL	D	24	1000000	121	6050000000	3.147132
LL	E	24	1000000	82	4100000000	2.391816
LL	F	24	1000000	102	5100000000	3.43105

Supplementary Table 1: population growth of fast- and slow- killing virus strains over 8 and 24 hours

© The Authors under the terms of the Creative Commons Attribution License http://creativecommons.org/licenses/by/3.0/, which permits unrestricted use, provided the original author and source are credited.
